# Editorial

**DOI:** 10.1093/bioadv/vbab001

**Published:** 2021-05-12

**Authors:** Alex Bateman, Thomas Lengauer

**Affiliations:** 1 Editors-in-Chief, Bioinformatics Advances; 2 Max Planck Institute for Informatics, Saarland Informatics Campus, Saarbrücken, Germany; 3 Institute of Virology, Faculty of Medicine and University Hospital Cologne, Cologne, Germany

Welcome to *Bioinformatics Advances*, an interdisciplinary journal on bioinformatics and computational biology. (In the following, we will use the term bioinformatics to stand for both bioinformatics and computational biology.) The journal represents a joint endeavor of the International Society for Computational Biology (ISCB) and Oxford University Press (OUP).

Why, with the many publication outlets available for computational biology research do we believe that there is a place for another journal on computational biology? Actually, there are quite a few reasons.

1. Bioinformatics is a still-growing field. The field has established its relevance in research in the life sciences in the past couple of decades. However, due to its interdisciplinary character, many of its contributions are spread over a wide variety of topical journals. This journal aims at strengthening the awareness of the central role bioinformatics plays in the life sciences within the scientific community. It does so by illuminating the field in its entirety and by providing a balance between methodical contributions and data analyses providing novel biological insights. The journal aims at providing a venue for many high-quality contributions to our field that, so far, are at the risk of disappearing in the vast landscape of topical journals.
2. Bioinformatics is not only growing but evolving, with new promising concepts and approaches continually appearing on the horizon, often based on new experimental technologies but sometimes also representing new paradigms in data analysis, modeling and simulation. *Bioinformatics Advances* aims at placing early spotlights on these emerging areas as the field develops.
3. We feel that there is much room to develop our interdisciplinary field as a community, as we strive for fairer representation of contributions from the diverse corners of our scientific community. Therefore, this journal will expend special efforts on accounting for the diversity of our contributing scientists, be it with respect to scientific background, gender, seniority, ethnicity or geographical region. In order to make the scientific contributions widely available, this journal will be fully open access and online only.

The journal’s goals spelled out above mirror the goals of the ISCB. In 2021, the ISCB enters its 25th year furthering the scientific field and the community that is its base. Over that time, the ISCB has had a strong working relationship with the publisher OUP. Examples include *Bioinformatics* being an official journal of ISCB today and for much of the lifetime of the society, the proceedings of ISCB’s flagship conference, the Conference on Intelligent Systems for Molecular Biology (ISMB) being published within *Bioinformatics* and OUP regularly sponsoring ISMB. Therefore, it is natural that OUP and the ISCB run this journal together.

Aside from original research contributions, the journal will offer formats that reflect the development of the field and its community, special collections and sections on important scientific meetings, reviews and opinion pieces as well as personal profiles of and perspectives by special contributors to our field.

The journal will maintain close connections to the journal *Bioinformatics* published by OUP. This entails the following aspects:

1. Both the scope and the article types of *Bioinformatics Advances* will include and expand those of *Bioinformatics.* With respect to scope, *Bioinformatics Advances* will reach beyond method-centered articles and include application-centered articles (Discovery Notes) and contributions to emerging areas as well as reviews, opinion and perspective articles. Furthermore, *Bioinformatics Advances* will include special feature articles that reflect the life of the scientific community, in general, and of the ISCB, in particular, such as personality features on recipients of prestigious awards in the field and, information on funding and policy and ethics and more.
2. Certain contributions that have been submitted to *Bioinformatics* but could not be accepted by that journal will be eligible for direct transfer to *Bioinformatics Advances.* Both journals will have compatible submission guidelines to enable simple and speedy transfer without the need for reformatting.
3. Transferring manuscripts with reviews from *Bioinformatics* will reduce the burden on reviewers of papers. In a similar vein, *Bioinformatics Advances* will also consider papers that have been reviewed previously for other journals. If the other journal will confirm the veracity of the reviews, our editors will take them into account in their decision-making.

We work with a two-tier editorial team. The team consists of a set of Associate Editors and of an Editorial Board. Our small team of Associate Editors will grow as the number of submissions increases. From the beginning, we place priority on the scientific excellence, editorial expertise and potential of our Associate Editors. At the same time, we look for broad topical coverage and for high diversity among the editorial team. The Editorial Board consists of established scientists who will help furthering the direction of the journal, reviewing specific papers and working out issues that arise.

We cordially welcome our inaugural editorial team that is listed below and thank all of them for their commitment to bringing this new journal to fruition.

We are happy and honored to welcome a stellar group of inaugural Associate Editors:

**Bissan Al-Lazikani, Institute of Cancer Research, London, UK**

Bissan Al-Lazikani's research focuses on developing and applying multidisciplinary computational techniques for drug discovery and clinical application. She is also Head of Data Science at the ICR, where she leads the Knowledge Hub Big Data Team for intelligent application of computers in adaptive, individualized therapy.

**Ivet Bahar, University Pittsburgh School of Medicine, USA**

Ivet Bahar is a Distinguished Professor and John K. Vries Chair, Department of Computational and Systems Biology at the University of Pittsburgh School of Medicine. Her research focuses on understanding the mechanisms of Biomolecular system interactions and their binding, catalytic and allosteric signaling effects. A major research goal of her lab is to investigate the dynamics of molecular systems in the cellular environment cellular using fundamental principles of physical sciences and engineering.

**Sofia Forslund, Max Delbrück Center for Molecular Medicine, Berlin, Germany**

Sofia Kirke Forslund is a computational biologist and a junior group leader at the Experimental and Clinical Research Center (ECRC), a joint venture between the Berlin Max Delbrück Center (MDC) and the Charité University Hospital. Her research group focuses on host–microbiome factors in cardiovascular diseases, working toward the goal of data-driven models for how human host and microbiome develop together under different conditions toward either health or disease.

**Marieke Kuijjer, Centre for Molecular Medicine Norway, University Oslo, Norway**

Marieke Kuijjer is a Group Leader at the Centre for Molecular Medicine Norway (NCMM), University of Oslo, Norway; adjunct Assistant Professor at Leiden University Medical Center (LUMC), the Netherlands. Her research focuses on developing computational frameworks that place genomic data into the context of gene regulatory networks and on exploring how these networks influence cancer and complex disease.

**Nicola Mulder, University of Cape Town, South Africa**

Nicola (Nicky) Mulder is a Professor and the Head of Division, Computational Biology, Department of Integrative Biomedical Sciences; Member of the Institute of Infectious Disease and Molecular Medicine (IDM), Faculty of Health Sciences, University of Cape Town. She also serves as the Principal investigator of H3ABioNet, a Pan African bioinformatics network for H3Africa. Her main research interests lie in the areas of genomics and its application to human diseases of relevance to Africa.

**Aïda Ouangraoua, Université de Sherbrooke, Canada**

Aïda Ouangraoua is an Associate Professor at the Department of Computer Science of Université de Sherbrooke where she holds the Canada Research Chair in Computational and Biological Complexity. She leads the Computational Biology (CoBIUS) Team at the Computer Science Department of Université de Sherbrooke. Her research focuses on developing algorithms and comparative genomics methods to study the evolution of genome structures, gene splicing structures and RNA structures.

**Alexandros Stamatakis, Heidelberg Institute for Theoretical Studies, Karlsruhe Institute of Technology, Germany**

Alexandros Stamatakis is the head of the Computational Molecular Evolution (CME) research group at the Heidelberg Institute for Theoretical Studies (HITS) and is the Chair in High Performance Computing in the Life Sciences (Department of Informatics) at the Karlsruhe Institute of Technology (KIT). His research interests are in algorithms, parallel computing, parallel architectures, computational phylogenetics and evolutionary bioinformatics.

**Zhang Zhang, National Genomics Data Center, Beijing Institute of Genomics, Chinese Academy of Sciences and China National Center for Bioinformation, Beijing, China**

Zhang Zhang is a Distinguished Professor and Associate Director of National Genomics Data Center (NGDC), Beijing Institute of Genomics, Chinese Academy of Sciences and China National Center for Bioinformation (CNCB). His research focuses on big data integration and curation, computational health genomics and computational molecular evolution.

It is our goal to make the journal as attractive to authors and readers as possible. We hope that you will join us on this new adventure through reviewing for and publication in *Bioinformatics Advances*.

